# The complete mitochondrial genome of *Lutjanus ophuysenii* and phylogenetic analysis

**DOI:** 10.1080/23802359.2021.1951140

**Published:** 2021-07-15

**Authors:** Peng Sun, Yazhou Jiang, Xingwei Yuan, Hui Zhang

**Affiliations:** Key Laboratory of East China Sea Fishery Resources Exploitation, Ministry of Agriculture, East China Sea Fisheries Research Institute, Chinese Academy of Fishery Sciences, Shanghai, China

**Keywords:** *Lutjanus ophuysenii*, mitochondrial genome, phylogenetic analysis, Lutjanidae

## Abstract

The complete mitochondrial genome of *Lutjanus ophuysenii* was analyzed by the next-generation sequencing. It was composed of 13 protein-coding genes (PCGs), 22 transfer RNA genes (tRNA), 2 ribosomal RNA genes (rRNA), and a control region with a total length of 16,498 bp. This mitochondrial genome has a base composition of 27.78% A, 16.55% G, 30.68% C, and 24.98% T. The phylogenetic analysis revealed that the *L. ophuysenii* has the closest relationship with *L. votta*. This mitochondrial genome provides important information for the phylogenetic relationship and genetic resource in this species.

*Lutjanus ophuysenii* (Bleeker 1860) belongs to the family Lutjanidae and genus *Lutjanus*. This family in the order of Eupercaria and Lutjaniformes comprises 17 genera and 185 species worldwide (Nelson 2006). The Genus *Lutjanus* is reported to distribute in the Yellow Sea, the southern East China Sea and the Pacific and Indian Oceans (Kim and Kim 2016), and often occurs in the coral reefs and mangrove habitats in tropical and subtropical waters. They are often valuable economic fish in their native areas and have important ecological service functions. Among them, *L. ophuysenii* is distributed throughout southern Japan, southern Korea, and the eastern and southeastern coasts of China. Due to its high similarity in appearance to *L. vitta*, *L. ophuysenii* had been synonymized under the latter by many researchers (Lee and Cheng 1996). Mitochondrial DNA (mtDNA) is an important model system for molecular evolution and phylogenetic study, and the complete mitochondrial genome is viewed as an excellent molecular marker for phylogenetic relationship study (Pan et al. 2021). In this study, the complete genome information of *L. ophuysenii* was provided for an in-depth study of its phylogeny and evolution.

A specimen of *L. ophuysenii* were collected from the central coastal area of Zhejiang in East China Sea (28.4830°N, 124.9670°E). This specimen and DNA sample extracted from it were currently deposited in the storage of the East China Sea Fisheries Research Institute, Chinese Academy of Fishery Sciences (https://www.ecsf.ac.cn/, Peng Sun, sunpeng0512@hotmail.com) under the voucher number LOP2020801001 and LOP2020801001-dna, respectively. The total genomic DNA was extracted from muscle via DNA Extraction Kit (Tiangen, Beijing), and mitogenome was sequenced through Illumina NovaSeq PE150 at the Shanghai Personal Biotechnology Co., Ltd. Then, the whole genome DNA was assembled by the SPAdes (Bankevich et al. 2012) and annotated by the MITOS (http://mitos.bioinf.uni-leipzig.de/) (Bernt et al. 2013).

The mitogenome of *L. ophuysenii* was a circular molecule (GenBank accession number: MZ042266) with the base composition of 27.78% A, 16.55% G, 30.68% C, and 24.98% T, exhibiting AT bias (52.77%). It was composed of 13 protein-coding genes, 22 transfer RNAs, 2 ribosomal RNA (12S rRNA and 16S rRNA), and a control region with a total length of 16,498 bp. All of these protein-coding genes start translation with initiation codon of ATG, except for ND6 (ATT). The termination codons were TAA for genes of ND2, ND4, ND5, ND41, COX1, ATP6, ATP8, COX3 and COB, and TAG for genes of ND1, ND3, ND6 and COX2.

To investigate the evolutionary relationship of *L. ophuysenii*, all of the 13 protein-coding genes were concatenated into a single sequence for phylogenetic analysis with species of Lutjanidae and Sparidae using maximum likelihood method (Figure 1) and a bootstrap value of 1000 replicates (Sudhir et al. 2016). Results showed that all fish species in genus *Lutjanus* grouped together, and fish in Sparidae formed another branch. In addition, *L. ophuysenii* present a closest phylogenetic relationship with *L. vitta*, which was consistent with result of Kim and Kim (2016). This study adds to the genetic resources available for *L. ophuysenii*, which can be used for taxonomic and genetic evolution research in this species.

**Figure 1.**
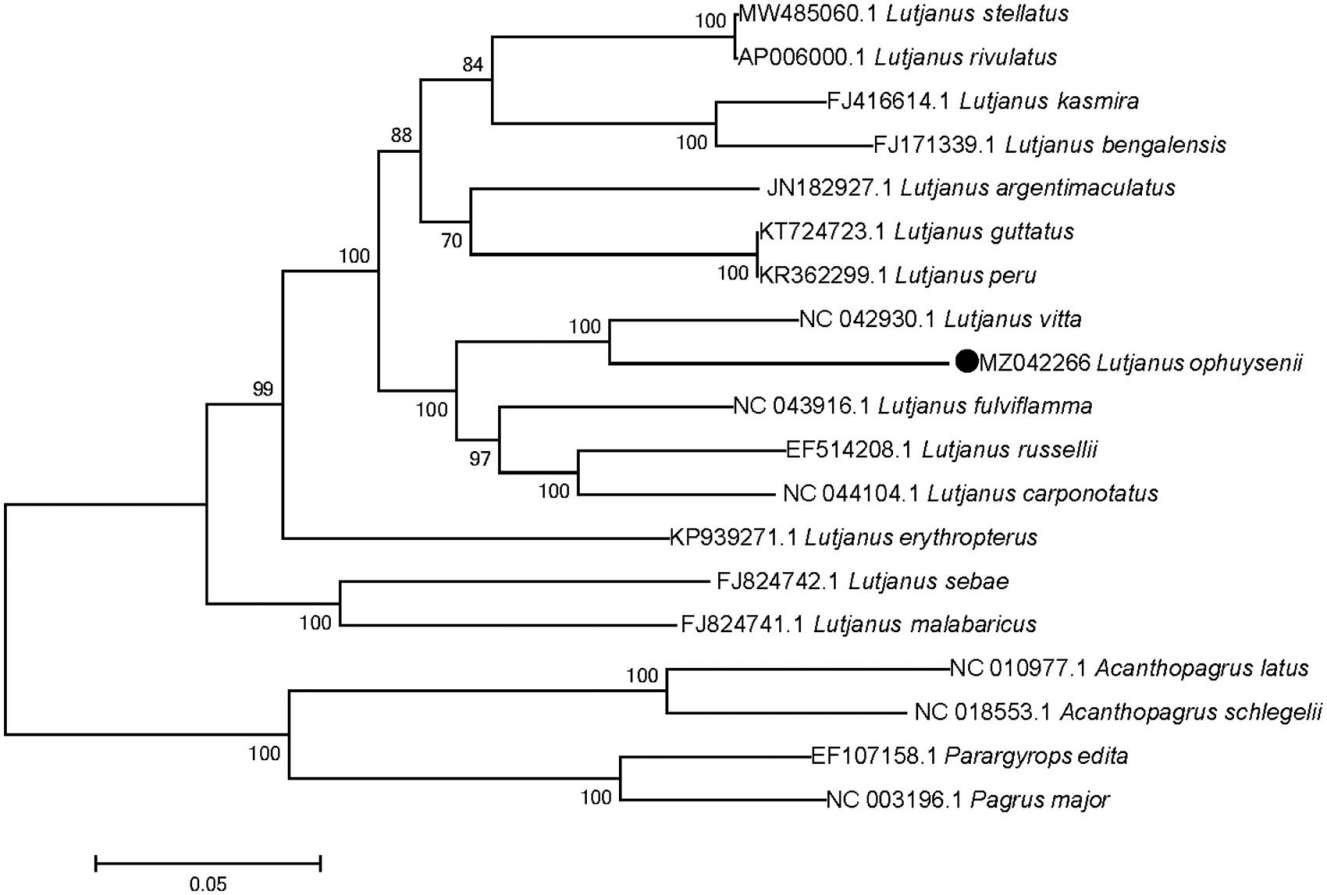
Phylogenetic tree reconstructed based on mitochondrial genome sequences from Lutjanidae and Sparidae. Analysis was using MEGA version 7.0 software with maximum likelihood method and bootstrap value of 1000 replicates.

## Data Availability

The data that support the findings of this study are openly available in GenBank of NCBI at (https://www.ncbi.nlm.nih.gov/) under the accession number of MZ042266. The associated BioProject, SRA and BioSample numbers are PRJNA724925, SRR14664913 and SAMN18865255, respectively.
